# Drug Development Digital Twins for Drug Discovery, Testing and Repurposing: A Schema for Requirements and Development

**DOI:** 10.3389/fsysb.2022.928387

**Published:** 2022-06-20

**Authors:** Gary An, Chase Cockrell

**Affiliations:** Department of Surgery, University of Vermont Larner College of Medicine, Burlington, VT, United States

**Keywords:** digital twin, drug discovery, drug repurposing, machine learning and AI, multiscale modeling, translational systems biology, *in silico* trials

## Abstract

There has been a great deal of interest in the concept, development and implementation of medical digital twins. This interest has led to wide ranging perceptions of what constitutes a medical digital twin. This Perspectives article will provide 1) a description of fundamental features of industrial digital twins, the source of the digital twin concept, 2) aspects of biology that challenge the implementation of medical digital twins, 3) a schematic program of how a specific medical digital twin project could be defined, and 4) an example description within that schematic program for a specific type of medical digital twin intended for drug discovery, testing and repurposing, the Drug Development Digital Twin (DDDT).

## INTRODUCTION

Medical Digital Twins are a hot topic, with a plethora of publications and opinions on what constitutes a medical digital twin (Björnsson et al., 2020; Croatti et al., 2020; Braun, 2021; Kamel Boulos and Zhang, 2021; Laubenbacher et al., 2021; Masison et al., 2021). The goal of this Perspective is not to say whether a particular use of the term is right or wrong, but rather put forth a program where the term is specifically defined for a particular use-case in a way that demonstrates fitness-for-purpose of the digital twin, what is hoped to be achieved, and how those factors affect how the digital twin is developed. Therefore, this Perspectives article has the following main aims:

1. Relay the properties of an industrial digital twin, which is the source of the concept.
2. Delineate specific features of biology that challenges the ability to meet the definition of a digital twin.
3. Describe a program for what it would take to develop a medical digital twin for drug discovery, testing and repurposing.

### Industrial Digital Twins: Definitions and Properties

A digital twin, as defined by the individual who introduced the concept, comprises of (Grieves, 2019):

1. A data structure for the real-world system
2. Some process that links data together to form dynamics
3. Some link to the real world that feeds back data into the data-propagation/generation process

The requirements for a digital twin have been expanded thusly (Wright and Davidson, 2020):

“In general, a model for a digital twin should be:

- sufficiently physics-based that updating parameters within the model based on measurement data is a meaningful thing to do,
- sufficiently accurate that the updated parameter values will be useful for the application of interest, and
- sufficiently quick to run that decisions about the application can be made within the required timescale”.

We note that the term “physics-based” is equivalent to “mechanistic”, meaning that the model underlying the digital twin is generatively causal. We also note, from the industrial digital twin literature that:

“It would also be possible to construct a purely data-driven model to sit at the heart of a digital twin. This approach is often not advisable for several reasons. The most obvious is that a data driven model is only reliable within the region of input parameter space from which the data used to construct the model was taken. Using data-driven models for extrapolation without imposing any constraints based on physical knowledge is a dangerous approach.”

(Wright and Davidson, 2020)

While the industrial use of digital twins has expanded as well, these appear to be core and conserved properties. The question is, what steps do the biomedical sciences need to take to fulfill these criteria?

#### How Biomedicine Generally Fails to Meet the Requirements for Industrial Digital Twins and What can Be Done to Correct That

The primary difference between industrial digital twins and potential medical digital twins is that in biomedicine the underlying specification is not known. Industrial digital twins are applied to objects and systems, for which, because they are engineered, there is a known formal specification. Furthermore, because the engineering task invariably involves applying constraints to a design task, the parameters of that specification and how alterations in those parameters precisely affect the dynamics of that specification are explicitly known. This is not the case in biology, where the “specification” needs to be reverse engineered through the process of basic biological research, with all the attendant uncertainties and insufficiencies in the resulting “product.” Note that this refers to representations of biology at the cellular and molecular level and for translational/clinical purposes; this does not apply to certain biological systems than can be reliably reduced to physics-based representations (such as material properties or fluid dynamics) or classical organ-level physiology (Bekiari et al., 2018; Jin et al., 2018). Because of this perpetual epistemic uncertainty, the formal specification that is the traditional basis for an industrial digital twin does not exist. This means that if medical digital twins are to be deployed the process of using them must account for uncertainties in their underlying specifications.

Intimately linked with the issue of lack of a unified formal specification is the issue of error propagation (Tellinghuisen, 2001). Engineered/industrial systems have the ability to quantify uncertainty (typically due to measurement limitations, but can also incorporate stochasticity) in the various features of their models. The ability to quantify uncertainty then leads to the ability to propagate “error” in the model at each time step, generating a constrained probability cloud of future system-level trajectories, which can then inform how often the digital twin needs to be updated with real-world data. In contrast, the biomedical sciences can often neither rigorously quantify their experimental error (Robert and Watson, 2015), nor can they strictly predefine the constraints on the range of potential system trajectories that arise from a perturbation (An, 2018).

We have proposed that sufficiently complex multi-scale mechanistic models can act as formal objects that instantiate specific unifying hypotheses (An, 2018); so too this concept can be applied to the development and use of “medical” digital twins. The epistemic uncertainty associated with biological systems leads to the concept of a medical twin “specification” as an ensemble of candidate specifications that need to be refined, modified or discarded through a continuous evaluation process. Therefore, there is an inherent discovery aspect to the deployment of a medical digital twin; the process of selection among specifications and accounting for unknown heterogeneity/stochasticity need to be included. For an example of how ongoing model refinement can be tied to concurrent model use we look to the case of hurricane prediction.

In the evolution of weather modeling the initial models were crude but it was readily recognized that for them to improve data collection and collection capability improvements were integrated into the overall endeavor. It was recognized that it would be advantageous to have the ability to forecast the trajectory of a hurricane well before the technological capabilities to do so were available. While computer modeling/forecasting of hurricane trajectories began in the 1950’s, it was not until the early 2000s, after decades of improvements both to the models as well as the computational infrastructure used to implement the models, that the models began to achieve a high level of fidelity, and even now, it is recognized that the computational forecasts must be periodically updated with real-world data to maintain their utility. The key here is that those responsible for collecting the data recognized the importance of these models, even in their infancy. Since having an ongoing data stream that links the physical world to the virtual one is intrinsic to the concept of a digital twin (Grieves, 2019; Wright and Davidson, 2020), this engagement of the data-generating community is critical.

### STEPS FOR DEVELOPING A MEDICAL DIGITAL TWIN: A GENERAL DESIGN SCHEMA WITH A SPECIFIC EXAMPLE OF A DIGITAL TWIN FOR DRUG DISCOVERY, TESTING AND REPURPOSING

**FIGURE 1 |:**
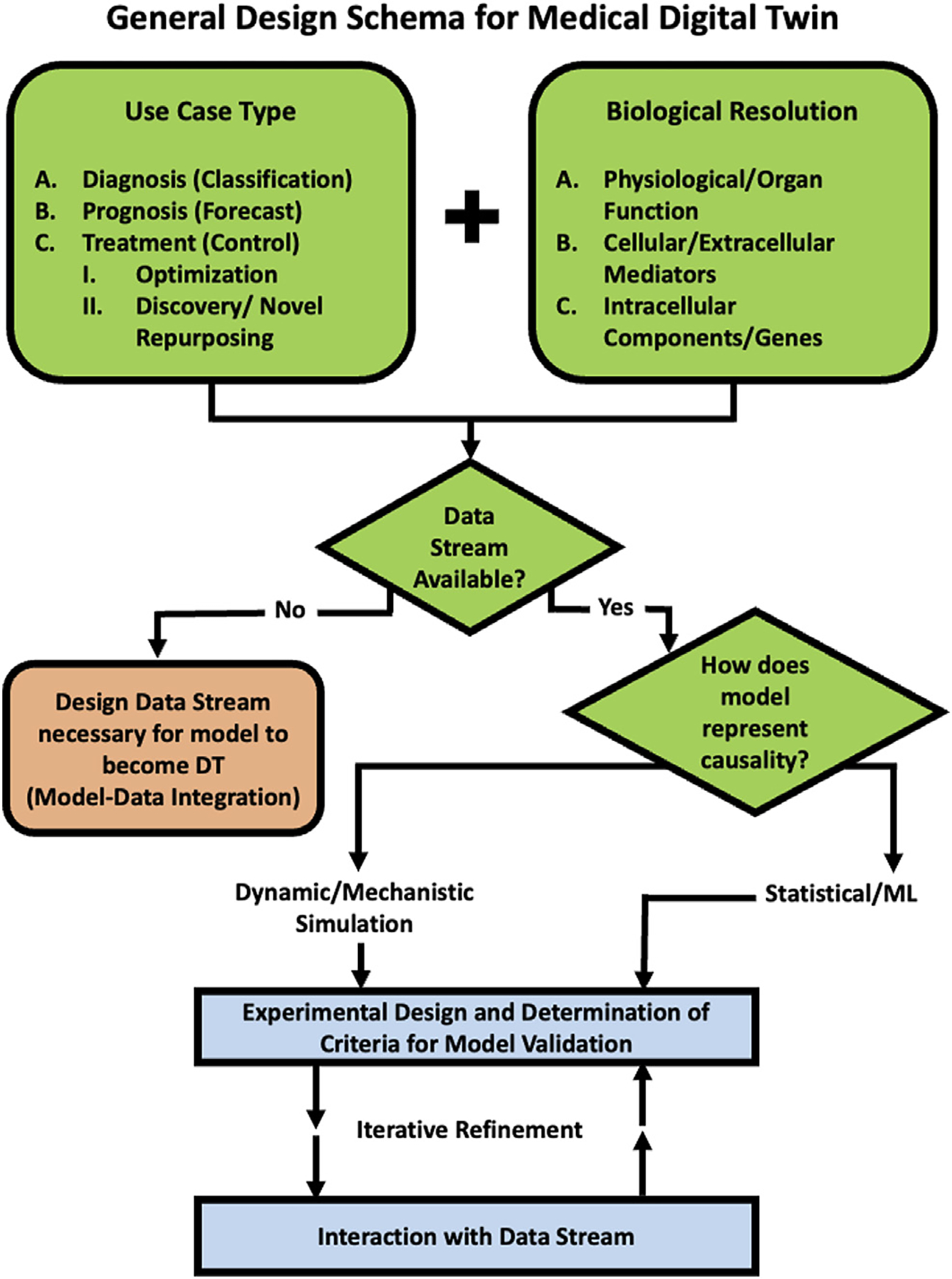
Schematic Program for developing a Medical Digital Twin. This provides a guide to a series of decisions that should be made when embarking on the development of Medical Digital Twin. An example of a specific use case for drug development, testing and repurposing can be seen in *Example Use Case: Development of Medical Digital Twins for Drug Development, Testing and Repurposing Section* and Figure 2.

Given the lack of standards as to what defines a medical digital twin, a project that embarks on creating medical digital twin necessarily involves providing this definition for the specific use-case. We have developed the following schema that can aid in more precisely determining the fitness of various specification and modeling methods based on the intended use of the medical digital twin (see Figure 1).

#### Example Use Case: Development of Medical Digital Twins for Drug Development, Testing and Repurposing

**FIGURE 2 |:**
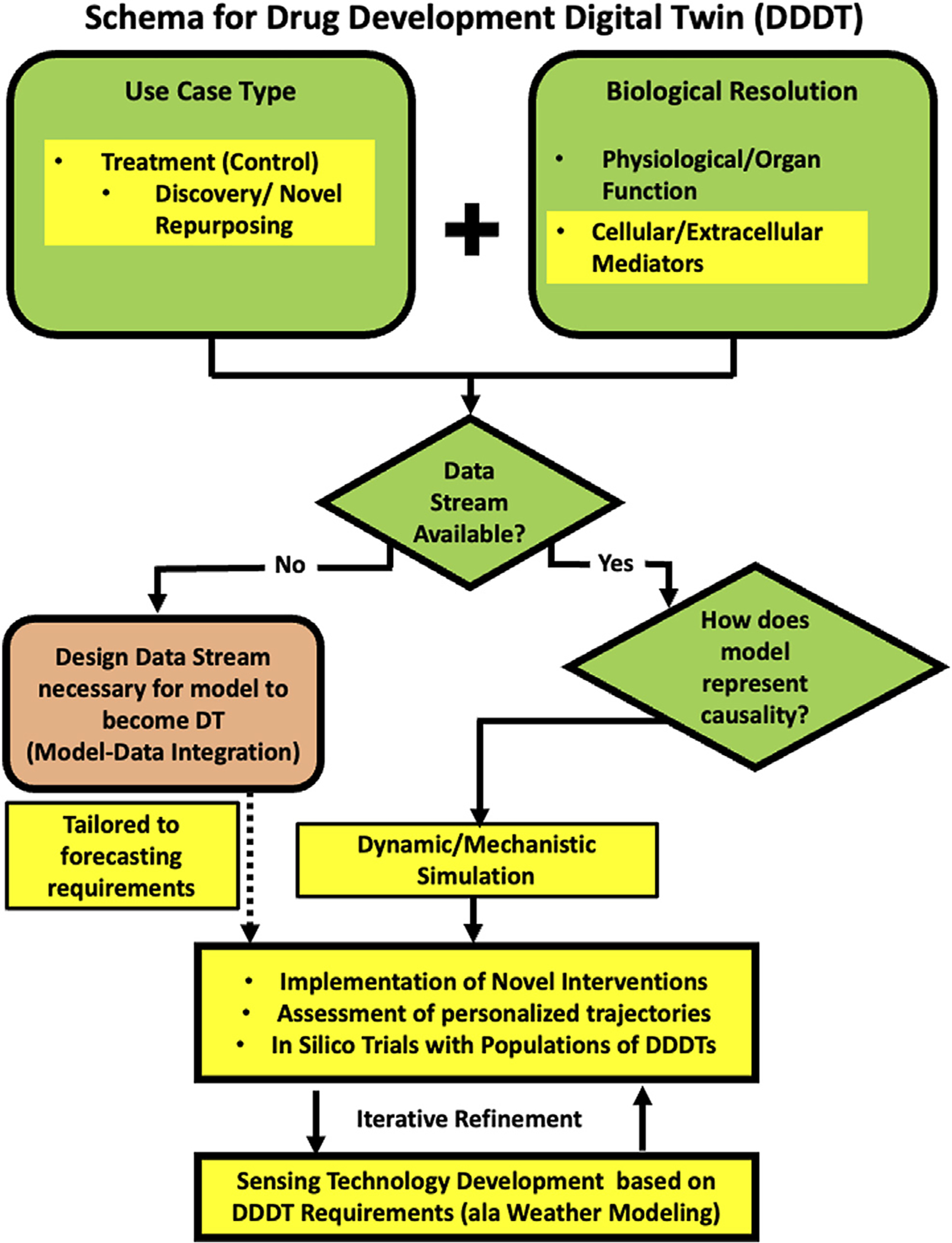
A schematic program for developing a medical digital twin for drug discovery, testing and repurposing, the Drug Development Digital Twin (DDDT). We adapt the general schema in Figure 1 to specific requirements for the task of discovering and evaluating novel therapeutic agents and/or novel applications of existing drugs in novel contexts. The unifying aspect of these tasks is that no prior clinical data exists because the interventions have not yet been tried. This results in the choices reflected by the Yellow Highlighted sections in the presented schema (compare to Figure 1 and see Text for details).

Here we describe a specific example of a development schema for medical digital twins with the expressed purpose of drug discovery, testing and repurposing; we term this class of medical digital twins as *Drug Development Digital Twins (DDDTs)*. The repurposing task is not about optimization of existing therapies for a particular disease; rather the drugs are being repurposed into a different context from which they have been found to be effective (e.g. the attempt to use monoclonal anti-cytokine therapies approved for auto-immune disease for the cytokine storm seen in acute viral infection). The emphasis on “discovery” places the capabilities of the DDDT in assessing the landscape of the unknown. As such, there is no pre-existing data related to certain tasks inherent to personalization as optimization: no demonstration of biological efficacy, no pharmacokinetics, no retrievable response rate of the drug for the disease. The key to this program is that we are seeking to answer what is the main goal of biomedical research: finding therapies that work. The following sections will provide an example of how the general schema depicted in Figure 1 informs how a DDDT can be described (applicable and critical answers listed in **bold** and an adaption of Figure 1 specific for a DDDT Schema in Figure 2).

##### Use Case Type

c. Treatment (Control Task)

ii) Discovery/testing of novel treatment (includes combination therapy of existing treatments and repurposing of existing treatments to new diseases

Comments:

- The purpose of the DDDT is to examine the potential effectiveness of novel drugs, hypothetical drugs, and existing drugs used in a new population for a new application. Therefore, this is not about optimizing existing therapies for populations in which those therapies are already being employed (e.g. precision oncology).
- As such, there is not pre-existing clinical data about how the target population responds to the particular proposed intervention.
- Therefore, a DDDT must incorporate some representation of the presumed mechanism for the drug/drug candidate being examined (see next section).

#### Biological Resolution (May Have More Than one)

1. Physiological (Vital Signs, Measurements of bulk/whole organ function (e.g. Cardiac output/metrics, Lung capacity/compliance, etc.)
2. Cellular and Extracellular Mediators
3. Intracellular components/genes

Comments:

- While all three of these levels of biological resolution would be beneficial, because the goal of the DDDT is to represent the actions of a potential drug, which for all intents and purposes, are characterized at the molecular/cellular response level, the DDDT should be developed at this level of resolution. This is because:
  - Inference of cellular/molecular interactions to organ-level, physiological behavior is subject to all the complexity and non-linearities that bedevil the current drug development pipeline (Translational Dilemma).
  - Extrapolation of gene regulatory and other intracellular signaling networks to cell population behavior is similarly unable to represent the variations of behavior across that population.
  - Gene expression data, for the most part, is too sparsely characterized with respect to the functional consequences and effects of the noted genes.
- Many current mathematical models of biological processes operate are too abstract to be useful for the task of the DDDT. For instance, a mathematical model of viral infection that has an aggregated term for “pro-inflammation” cannot be used to evaluate the effect of a specific drug because “pro-inflammation” is not a mechanistic target for a chemical compound. This is analogous to the limitations of physiological scale models for the purpose of the DDDT.
- Therefore, DDDT models will be invariably more complex than many of the current models, which are often limited by concerns about finding the appropriate parameters. We address the challenge of parameterizing DDDT models using different ML/AI methods (Cockrell and An, 2021; Cockrell et al., 2021).

#### Data Feeds Available? Part of the Definition of a Digital Twin is That the DT is Updated With Data From the Real/Physical World

a. No

1. Your project is to design what data stream is necessary to turn your model into a digital twin.

Comments:

- Given the current state of being able to establish trustworthy formal representations of relevant biology the biomedical community finds itself in the same situation as the weather prediction community in the 1950s. Therefore, as mentioned above, intrinsic to a program for developing a DDDT is recognizing the importance co-development of data-acquisition and simulation modeling. This is represented in Figure 2 by the dashed arrow connecting a newly designed data stream to the Experimental Validation step.

b. Yes

i) Timescale/Interval?

1. How does your model link data points in a time series?
  - Statistical/ML
  - Dynamic Model/Simulation

Comments:

- While there may be a role of Statistical/ML correlative methods in aiding in the analysis, calibration and parameterization of the dynamic, mechanistic models ((Cockrell and An, 2021; Cockrell et al., 2021)), the previously noted fact that for the discovery task of the DDDT, sufficient data for the effective use of correlative methods almost certainly does not exist.
- Integrative methods exist that can help address the challenges of error propagation in the dynamic behavior of biological systems and their clinically relevant representation with mechanism-based simulation models underlying DDDTs (Cockrell and An, 2021; Cockrell et al., 2021).

Comments:

- Data collection should focus on obtaining data at the same level of biological resolution implemented in the mechanism-based models in the DDDT; this is necessary to effectively calibrate, parametrize and validate the core specifications of the DDDTs.
- Data at the higher-level physiological data also needs to be collected concurrently; this will allow the calibration and validation of the mappings between generative mechanisms and clinical phenotypes produced by the mechanism-based models in the DDDTs.
- Part of this integration process is defining and specifying methods and metrics for validation, including the design of new data streams needed to evaluate the DDDT (see Figure 2).

#### Existing Computational Models/Modules

Comments:

- There are two primary barriers that limit existing computational models and modules from being utilized as DDDTs: 1) they are not typically inter-compatible without significant software engineering efforts, rendering systems-level simulations difficult or low-resolution; and 2) the focus at present is typically on separating “the signal from the noise,” and in doing so, washing away complexity that leads to biological heterogeneity. Both of these challenges are readily addressable:
- Towards the first challenge, there has been extensive work on developing mechanism-based simulation models on various pathophysiological processes, and the critical issues regarding model sharing, credibility assessment and reuse have been recently cataloged in (Saccenti, 2021; Karr et al., 2022). The importance of model repositories and consortia development have been discussed in other papers concerning medical digital twins (Björnsson et al., 2020; Laubenbacher et al., 2021; Masison et al., 2021)
- Addressing the second challenge is necessary for the “Discovery” portion of the DDDT. The translational purpose of DDDTs also requires a shift in the goals of calibration and parameterization, namely that rather than trying to reduce complexity and heterogeneity, such tools applied to DDDTs need to embrace these features (Cockrell and An, 2021; Cockrell et al., 2021). The reason for this is the need to represent any individual requires the ability to represent every individual.
- If this can be achieved, then the discovery tasks inherent in the DDDT involves first the performance of *in silico trials* that consist of using populations of DDDT for either drug/control discovery or repurposing evaluation. These discovery tasks represent complex control problems, for which traditional control methods may not be sufficient and requires novel approaches for complex, multi-modal, adaptive control (which includes the integration of AI methods with mechanistic simulations (Ozik et al., 2018; Petersen et al., 2019; Larie et al., 2022).

#### Iterative Refinement

Comments:

- Iterative refinement of the DDDT will incorporate reverse engineering specifications of the biological system that are general enough to represent the range of human physiological and pathophysiological behaviors. This integrates the DDDT with existing basic science research, but is able to integrate that research expressly for translational and clinical purposes.
- While the data collection should be structured by the needs of the models within the DDDT, the collection and analysis of biological samples is the largest, most complicated, and most limiting aspect of this project.
  - In an ideal world the frequency of sampling would be consistent during the entire disease course; in reality such time series invariably too sparse to capture biological behavior. There will need to be a means to intensify sample collection interval if need is identified by DDDT simulation experiments.
  - As with the weather model evolution paradigm, information generated with simulation experiments with the models underlying DDDTs would ideally spur the development of bedside/point-of-care/real-time multiplexed mediator or cell-population assays, and the engagement need to engage aspects of industry working on these technologies.
- Defining validation criteria is crucial, though the personalized nature of DDDTs may require novel approaches for determining adequacy of performance. For instance, forecasting the range of counterfactual possibilities for a complex therapeutic regimen is an inherent capability of the DDDT, but being able to have corresponding real-world data for such a policy on an individual/personalized basis (the ostensible rational for a medical digital twin) is likely impossible. Overall assessment may require the execution of *in silico* trials consisting of virtual populations of DDDTs (An, 2022), for which more “traditional” statistical measures of efficacy can be used. Alternatively, the “personalization” of DDDTs may require generating “forecasting cones” during an initialization period (Larie et al., 2021) in the equivalent of a Phase 1 safety trial for any patient-care technology utilizing the DDDT.

## CONCLUSION

We introduce a general schema that can provide guidance in the development of a medical digital twin, and herein provide an example of how that schema can be used to focus on key aspects associated with a particular type of medical digital twin: one developed for the explicit purpose of discovering what potential drugs might work, in silico testing of those potential drugs and in silico testing of existing drugs repurposed for a completely different disease, the DDDT. Specifically, we pose several requirements for a DDDT:

1. Since the interventions are novel (either new agents or new contexts) the underlying model must be a mechanistic simulation (i.e. there is no pre-existing clinical data for a statistical/ML model).
2. The underlying simulation model should represent the putative mechanisms of the potential therapies being evaluated.
3. Data streams will have to be designed and refined guided by the requirements of the underlying simulation model (ala weather prediction).
4. Evaluation of the validity and eventual trustworthiness of DDDTs will require a combination of traditional statistical evaluation (i.e. efficacy as assessed *in silico* clinical trials) and novel means for assessing personalized forecasting and counterfactual characterization.

While DDDT portrayed above is not currently achievable, we consider the description above an aspirational roadmap to transition the current paradigm(s) in computational biomedical modeling from useful abstractions into generalizable translational and clinical tools, i.e., DDDTs. This requires an evolution both in the way biomedical computational models are considered and developed, with a necessary focus on representing biomedical heterogeneity, and how models can drive the way that biomedical data is collected with the goal of iteratively informing and refining DDDT models, such that high-fidelity, multi-scale, system-level representations of human patients can be calibrated and validated to a level of trust corresponding to the formal specifications that underlie Industrial Digital Twins.

## Data Availability

The original contributions presented in the study are included in the article/Supplementary materials, further inquiries can be directed to the corresponding author.
